# Pilot Study of Trans-oral Robotic-Assisted Needle Direct Tracheostomy Puncture in Patients Requiring Prolonged Mechanical Ventilation

**DOI:** 10.3389/frobt.2020.575445

**Published:** 2020-11-27

**Authors:** Xiao Xiao, Howard Poon, Chwee Ming Lim, Max Q.-H. Meng, Hongliang Ren

**Affiliations:** ^1^Department of Electrical and Electronic Engineering, Southern University of Science and Technology, Shenzhen, China; ^2^Department of Biomedical Engineering, National University of Singapore, Singapore, Singapore; ^3^National University of Singapore (Suzhou) Research Institute, Suzhou, China; ^4^Department of Otolaryngology-Head and Neck Surgery, National University Hospital, Singapore, Singapore; ^5^Department of Otorhinolaryngology-Head and Neck Surgery, Singapore General Hospital, Singapore, Singapore

**Keywords:** tracheostomy, minimally invasive surgery, flexible mini-robotic system, robotic needling technology, mechanical ventilation

## Abstract

COVID-19 can induce severe respiratory problems that need prolonged mechanical ventilation in the intensive care unit. While Open Tracheostomy (OT) is the preferred technique due to the excellent visualization of the surgical field and structures, Percutaneous Tracheostomy (PT) has proven to be a feasible minimally invasive alternative. However, PT's limitation relates to the inability to precisely enter the cervical trachea at the exact spot since the puncture is often performed based on crude estimation from anatomical laryngeal surface landmarks. Besides, there is no absolute control of the trajectory and force required to make the percutaneous puncture into the trachea, resulting in inadvertent injury to the cricoid ring, cervical esophagus, and vessels in the neck. Therefore, we hypothesize that a flexible mini-robotic system, incorporating the robotic needling technology, can overcome these challenges by allowing the trans-oral robotic instrument of the cervical trachea. This approach promises to improve current PT technology by making the initial trachea puncture from an “inside-out” approach, rather than an “outside-in” manner, fraught with several technical uncertainties.

## 1. Introduction

Tracheostomy, which creates an external opening to the trachea from the neck, is the most common surgical procedure performed in patients with respiratory diseases (e.g., COVID-19) needing prolonged mechanical ventilation (Hur et al., 2020; Mecham et al., 2020; Vargas et al., 2020). Other patients requiring this procedure frequently suffer from blockages in the upper airway, which may be due to the following reasons: subglottic stenosis, neck fractures, and presence of tumors in the head and neck (Gill-Schuster, 2016; Mieth et al., 2016). Other tracheostomy applications include helping comatose patients expel secretions from their upper respiratory tract or, in the long term, for patients with severe chronic conditions, such as obstructive sleep apnea (Fray et al., 2018). Currently, there are two methods of tracheostomy: Open Tracheostomy (OT) and Percutaneous Tracheostomy (PT).

OT is also called a surgical tracheostomy, which involves making a horizontal or vertical incision approximately half-way between the cricoid cartilage and sternal notch. Dissection is then carried down through the subcutaneous tissues and the platysma muscles, creating a window between the second or third tracheal ring to allow the insertion of a tracheostomy tube for ventilation (Sanji et al., 2017; Kidane and Pierre, 2018). A shoulder roll may also position the patient with an optimal neck extension. Although OT allows excellent visualization of the surgical field and structures, it results in a risk of infection and bleeding, making it an unpopular choice among operators. In surgical tracheostomy, the incidence of local hemorrhage or stomal infection was around 37% (Cipriano et al., 2015; Hoseini et al., 2018).

While OT is still considered the gold standard, PT has proven to be a feasible minimally invasive alternative, gaining attention in recent years (Kannan et al., 2017; Rashid and Islam, 2017). PT remains an attractive option because of the ease of the procedure in carefully selected patients. It also avoids the transfer of critically ill patients to the operating room, implying stresses on the heavily utilized ICU setting resources. PT typically makes a small incision through which a guiding wire is advanced under direct bronchoscopic visualization, resulting in lesser bleeding and infection. The incision is then dilated using dilators until it is wide enough to fit the tracheostomy tube (Sandor and Shapiro, 2016).

However, there are risks involved, and no consensus on which techniques (OT or PT) minimizes complications in critically ill patients (Johnson-Obaseki et al., 2016). Although PT's complication rate is lower than that of OT, PT is more likely to cause serious and permanent complications (Guinot et al., 2012; Simon et al., 2013). It is hard to locate the drilling position as operators can only estimate from anatomical laryngeal surface landmarks. Only 9 out of 20 of the catheters entered the trachea in the correct space between the first and second cartilage rings with external punctures done blindly. One-third of 20 catheters punctured the thyroid, based on a study conducted on cadavers (Dexter, 1995).

Percutaneous procedures posed significant clinical and technical challenges due to the confined workspace, complex surrounding anatomical structures, constrained access to the surgical site, and incomplete exposure and visualization of the surgical field. As a result, complications, such as perforation of the esophagus, considerable vessel injury, the cricoid fracture may be encountered in PT, whereas these complications are not often in OT.

There remains an unmet need to develop novel percutaneous procedures that improve safety outcomes and allows better visualization for the primary puncture, though PTs are routine procedures. Current challenges associated with the use of PT can be summarized as below.

1. A technician manually drives the introductory needle through tissue and cartilage. By manually driving the needle through tissue and cartilage, the technician risks puncturing through the trachea and esophagus in case of excessive force from the needle. This excessive force may lead to inadvertent perforation of the posterior trachea wall, and even the cervical esophagus, resulting in pneumomediastinum (Khandelwal et al., 2017).
2. No guiding of aligning mechanism as the needle actuates through tissue. The absence of a guiding aligning mechanism may result in blood vessels being damaged and causing fatal bleeding. Typically, the source of bleeding is from the anterior jugular venous system, which, if encountered, is ligated and divided. Small venous branches can be a continued source of intraoperative and post-operative bleeding (Pilarczyk et al., 2016; Kruit et al., 2018; Sasane et al., 2020).

Besides, intubation and tracheotomy will produce an aerosol in treating pneumonia, which poses a great cross-infection risk to the medical staff.

The da Vinci surgical robot's success has proved that robotic-assisted minimally invasive surgery has great advantages over traditional surgery, such as less trauma, fewer complications, reduced hospital time, and improved surgical outcomes (Li et al., 2020). During the last decade, many specialized surgery robots with different functions have been developed. However, studies on robot-assisted intubation and tracheotomy are rare (Gu et al., 2019; Xiao et al., 2020). Hemmerling et al. (2012) presented the first human testing of a robotic intubation system called the Kepler intubation system for oral tracheal intubation. Do et al. (2016) proposed a mechatronic tracheostomy tube for automated tracheal suctioning. Wang et al. (2018) developed a remote robot-assisted intubation system to improve the success rate of the pre-hospital intubation and rescue model. Most of the studies on the tracheotomy system localize the position between the endotracheal tube and the carina. To the best of our knowledge, there is no tracheotomy robot reported in the literature.

To improve the current PT procedures and to reduce the infection risk of the medical staff in infectious disease unit, we proposed a flexible mini-robotic system incorporating a robotic needling technology, thereby allowing precise access to the cervical trachea. By creating the first puncture using an inside out technique, we believed the PT's current challenges could be potentially addressed. The proposed flexible mini-robotic system will provide new capabilities in the following areas: accurate identification of the proposed trachea puncture, controlled force to the introductory drill, and precision robotic needling puncturing technology as it actuates through tissue into the neck. No device is currently available on the market for performing internal-to-external (i.e., inside-out) punctures through multiple layers of tissue and cartilage. Based on our evaluation, there is presently no development of similar trans-oral tracheotomy puncture systems. This method could provide the basis for other percutaneous, for example, placement of suprapubic catheters and nephrectomies and cholangiostomies.

## 2. Materials and Methods

Both endotracheal intubation and tracheostomy are used to improve respiratory function and facilitate ventilation support treatment. In endotracheal intubation, a flexible plastic tube is placed into the trachea through the patient's mouth. In tracheostomy, an opening is created on the patient's neck to place a curved tube.

By designing a robotic system that drills the hole from within the trachea and outwards, operators can see the drilling position using a camera. This system can cause fewer complications on the patient's neck and tracheae, such as excessive bleeding and the esophagus's possible damage. The proposed procedure is a combination of the endotracheal intubation and PT, which improves the current PT.

The size of the endotracheal tube and tracheostomy tube varies with age and gender. The commonly used measures for adults are listed in Table 1. The length from mouth to the trachea is about 21 and 23 cm long for female adults and male adults, respectively (Varshney et al., 2011). The trachea's average diameter for obese and non-obese adults is ~2.1 and 2 cm, respectively. The puncture should be performed between the first and second or between the third and fourth tracheal rings (Al-Ansari and Hijazi, 2006; Lerner and Yarmus, 2018). These parameters provide a reference for our design.

**Table 1:** Size of the endotracheal tube and tracheostomy tube.

**Instrument**	**Diameter (female/male mm)**	**Length (cm)**
Endotracheal tube	7.5/8.0	21–23
Tracheostomy tube	10/11	8

### 2.1. Design of the Flexible Robotic Needling System

**Figure 1:**
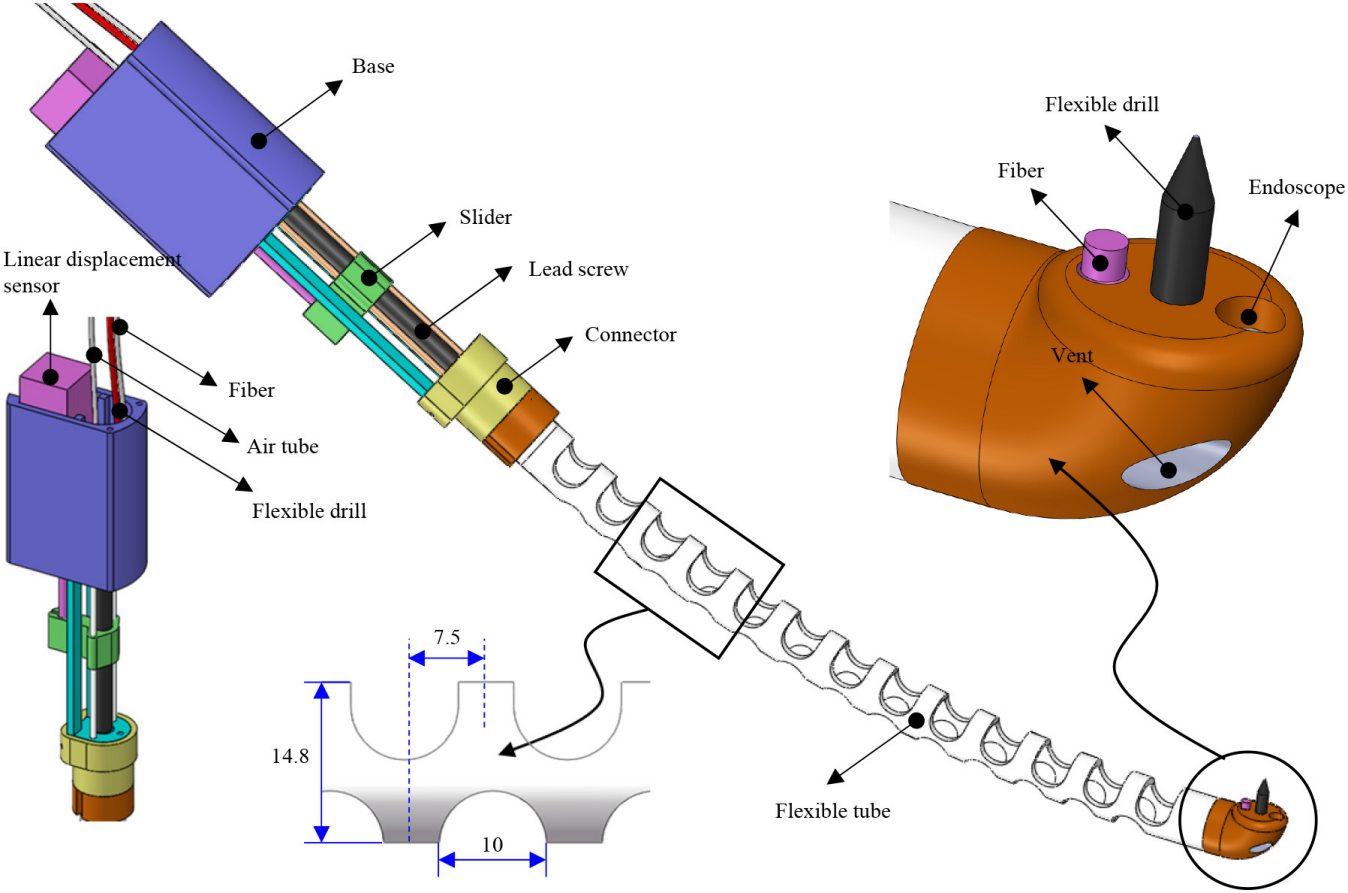
3D model of the flexible robotic needling system.

A compact and miniature mechanism with variable curvature should be designed to deploy the flexible drill through the mouth to the trachea. The mechanism based on the traditional joint is composed of too many parts, and the rigid structure is not the first choice for safety consideration. Compliant mechanisms are usually monolithic, which transmit motion and force through the elastic deformation of the flexures (Xiao and Li, 2016; Xiao et al., 2017). Combining the concept of compliant mechanism with soft robot and continuum robot, we designed a flexible tube. A flexible tube is designed. To realize the tans-oral tracheostomy puncture, a flexible robotic needling system is proposed, as shown in Figure 1. The flexible robotic needling system consists of a linear displacement modular, a flexible tube, and an end tip.

The flexible tube has one bending degree of freedom (DoF); it has an outer diameter of 14.8 mm, length of 180 mm, and 2 mm thickness. The uniformly arranged notches are adopted to increase the tube's compliance to be blended smoothly. Besides, the notches can also let air into the trachea. At the end of the tube, there is a rounded tip. The tip has five channels: flexible drill channel, camera channel, and fiber optic/tendon channel, respectively. The rests located on both sides are stabilization balloon channels, which decrease the vibration induced by the flexible drill. The tip's end surface is parallel to the centerline of the flexible tube so that the flexible drill can drill out vertically to the trachea. To control the bending of the flexible tube, tendon-driven is adopted. One end of the tendon is connected to the round tip, and another end is connected with the slider of the linear displacement modular.

**Figure 2:**
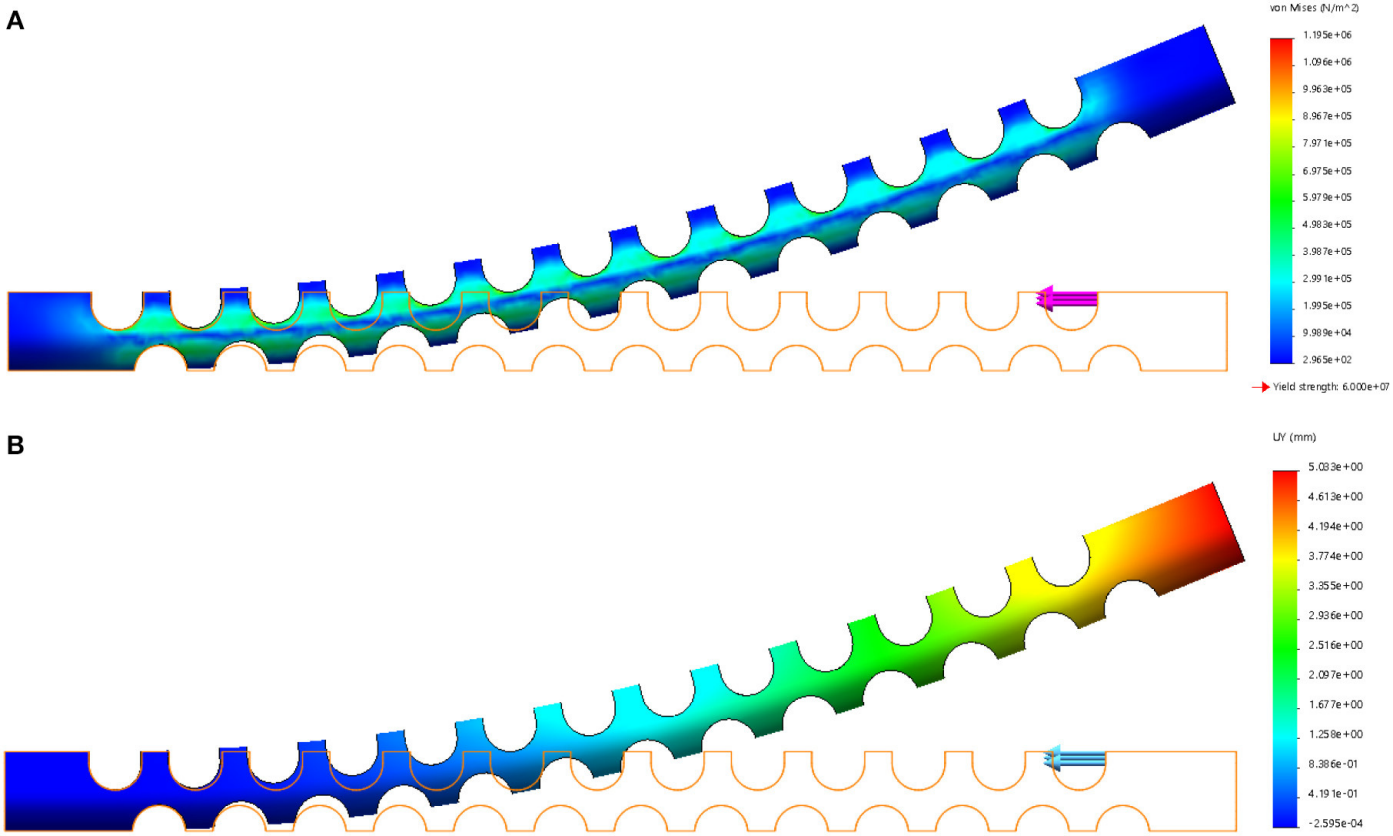
FEA analysis of the flexible tube. **(A)** Strain results, **(B)** Displacement results.

FEA verification is carried out by using the Solidworks Simulation add-ins to validate the flexible tube's compliance. The left side of the flexible tube is fixed, and a 1 N force is applied at the right upper side of the flexible tube. The result is shown in Figure 2, the strain distribution is illustrated in Figure 2A, it can be observed that the strain mainly occurs around the notches. This is due to the local stretch (bottom layer) and compression (upper layer) during the bending. The displacement of the flexible tube is shown in Figure 2B. The largest displacement is 5.033 mm. The compliance can be calculated as 5.033 mm/N and used as a reference for choosing the tendon.

**Figure 3:**
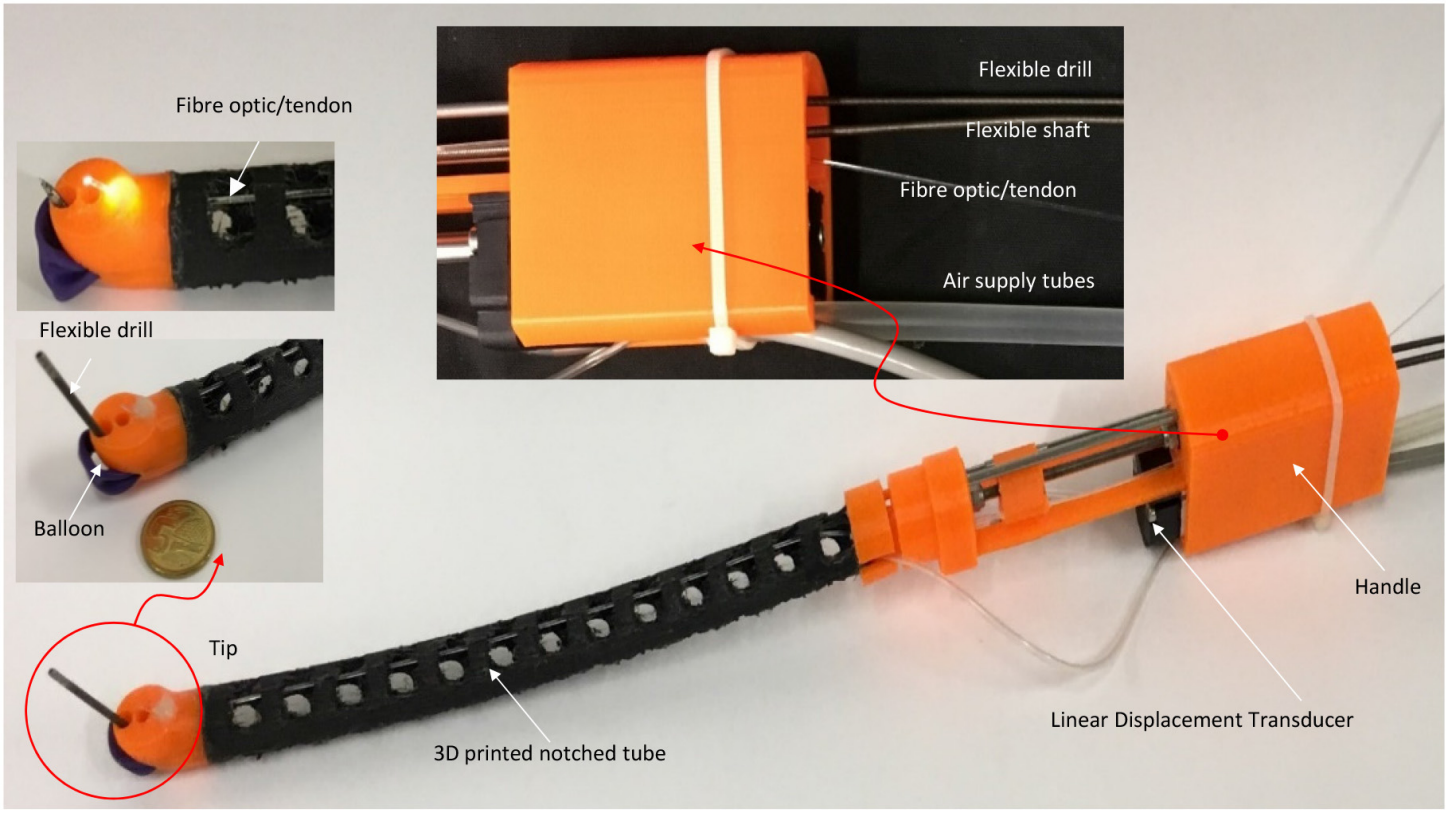
Prototype of the flexible robotic needling system.

A 3D printed prototype of the flexible robotic needling system is developed, as shown in Figure 3. NinjaFlex (NinjaTek, Manheim, PA, USA) is chosen as the flexible tube material due to its superior flexibility and longevity compared to other non-polyurethane materials. The end tip is 3D printed with 3D printed PLA (Poly Lactic Acid).

The flexible drill is a sharped flexible shaft (Hagitec Co Ltd., Tokyo, Japan). The flexible shaft is made of hard steel wires adhesively coiling incrementally thicker steel around a central rod in alternating directions. It can transmit adequate torque to the drill tip. The flexible shaft's helix coils can provide thrust force like a screw when it is rotating.

Fiber optic/tendon (0.75 mm diameter) is introduced to bend the tube and illuminate the visual field. The flexible drill, fiber optic/tendon, and air supply silicone tubes are housed in the flexible tube's cavum. The ends are connected to a DC motor, the linear displacement modular, and a DC mini electric air pump.

### 2.2. Overview of the Robotic-Assisted Trans-oral Tracheostomy System

**Figure 4:**
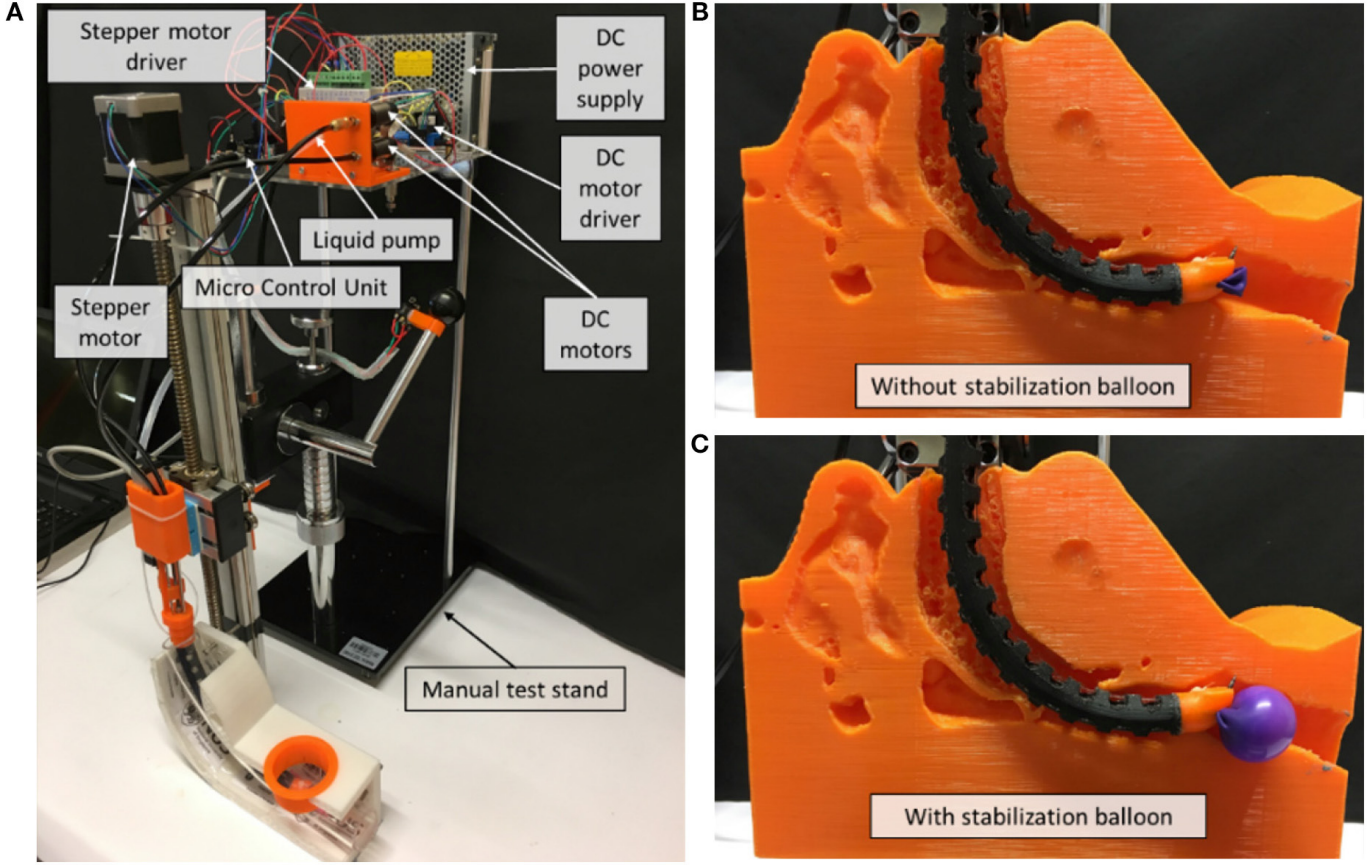
Overview of the robotic system. **(A)** System overview, **(B)** Working state without stabilization balloon, **(C)** Working state with stabilization balloon.

As shown in Figure 4, a prototype of the flexible mini-robotic tracheostomy system, is developed. The proposed system includes the following parts: a flexible robotic needling system, a stepper motor actuated linear displacement modules, a motion and control unit, DC power supply, and a manual test stand to support them. The flexible robotic needling system is fixed on the linear displacement module platform to pass through the curved oral cavity to the trachea. The working states of the system are demonstrated in Figures 4B,C.

The microcontroller used for our prototype is Arduino Due, which will be responsible for controlling the linear displacement module, two DC motors, and the air pump in controlling the vertical linear displacement of the prototype, actuating the tendon to achieve bending of the 3D printed notched tube, the drilling of the flexible shaft, and inflating the stabilization balloon, respectively.

**Figure 5:**
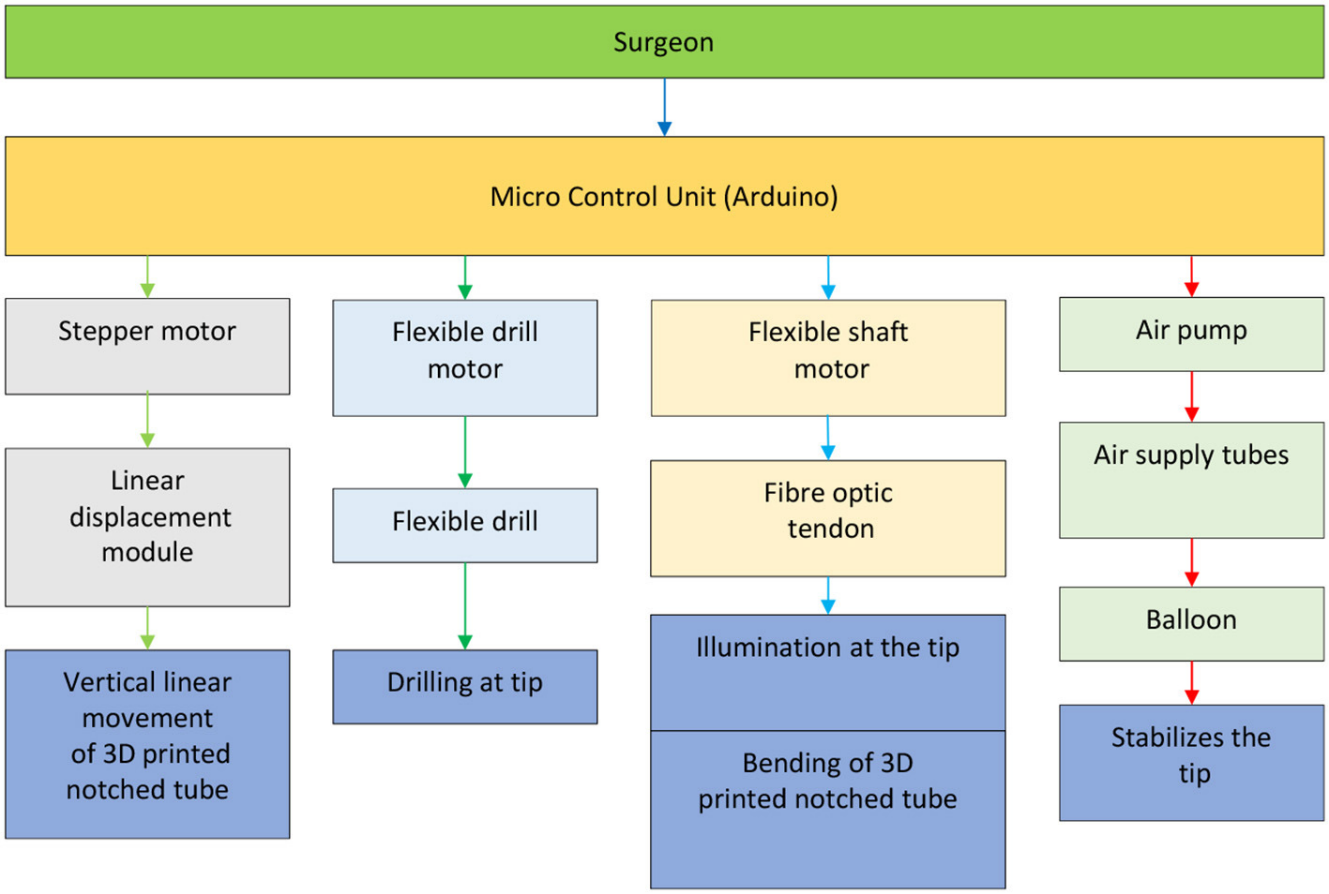
The control diagram of the prototype.

The control diagram for the prototype, shown in Figure 5, is made up of relatively independent modules, which primarily guarantees the robustness and convenience of use. It can work in a passive or automated driven way under compliance control. Human visual feedback enables the closed-loop control of 3D printed notched tubes in adjusting the flexible drilling shaft position and orientation. The operator can control the puncture speed. The fiber optic/tendon displacement is real-time recorded by the linear displacement transducer (Teed KTM-V2-25 mm, Dongguan Jingbiao Electronic Technology Co. Ltd, China) it will not exceed the limit.

Our proposed design is to incorporate the following novel solutions: (1) a flexible variable curvature tube with a flexible drill; (2) an illumination source and a tendon mechanism to the point of interest; and (3) stability in drilling. The illumination incorporates an optic fiber, which also acts as a tendon in our design. This illumination allowed us to pinpoint precisely the drilling location as the optic fiber near the point of drilling. The balloon stent mechanism is to provide stability when drilling. The exact amount of air can be controlled, and the balloon's pressure can be controlled. Therefore, the amount of force exerted in the trachea wall can be controlled to ensure the least amount of discomfort.

## 3. Results

### 3.1. Optic Fiber/Tendon Tensile Test

**Figure 6:**
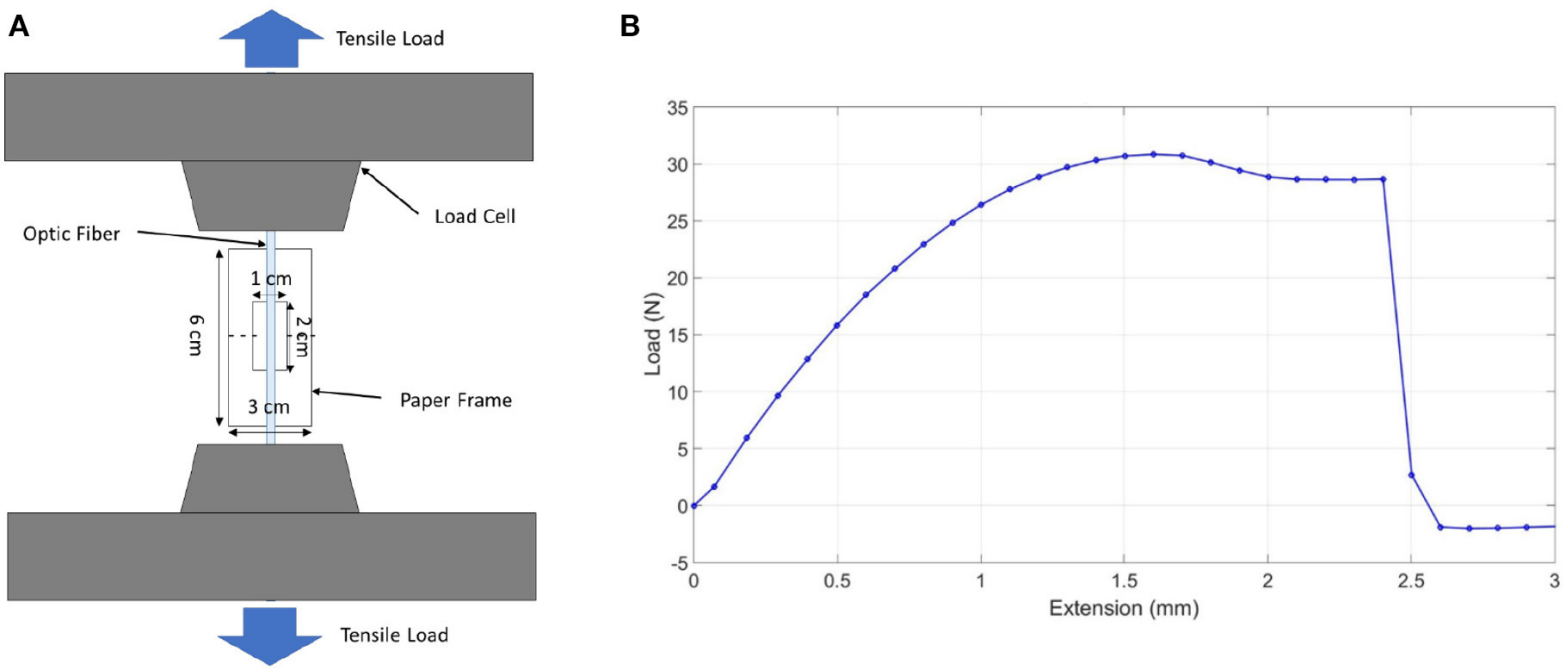
Optic fiber tensile test. **(A)** Monofilament/thread tensile test setup. **(B)** Optic fiber tensile test graph.

To measure the ultimate tensile strength of the fiber optic/tendon of the prototype, the monofilament/thread setup, shown in Figure 6A, was used. In this test, the optic fiber was placed over the center of the paper frame's slot with one end temporarily fixed with adhesive tape. Next, the same procedure was done to the other end of the optic fiber. A drop of superglue was then applied at both ends of the slots, which ensured that the optical fiber bonds firmly with the paper frame. The optic fiber, together with the frame, was then mounted onto the Instron machine. Before any load was applied, both sides of the paper frame were cut or burnt at mid-gauge (dotted line) with the paper frame unstrained. As shown in Figure 6B, the maximum tensile load the optic fiber could withstand, the tensile stress at full load experienced by the optical fiber, and the tensile strain at maximum load experienced by the optical fiber were 30.85 N, 120.88 MPa, and 0.05, respectively. According to the FEA analysis, the compliance of the flexible tube is 5.033 mm/N. A displacement of 50.33 mm can be achieved when 10 N force is applied at the end tip. Therefore, the fiber optic used was able to withstand the tension required to produce the prototype's bending motion.

### 3.2. Bending Without Load and With Load

**Figure 7:**
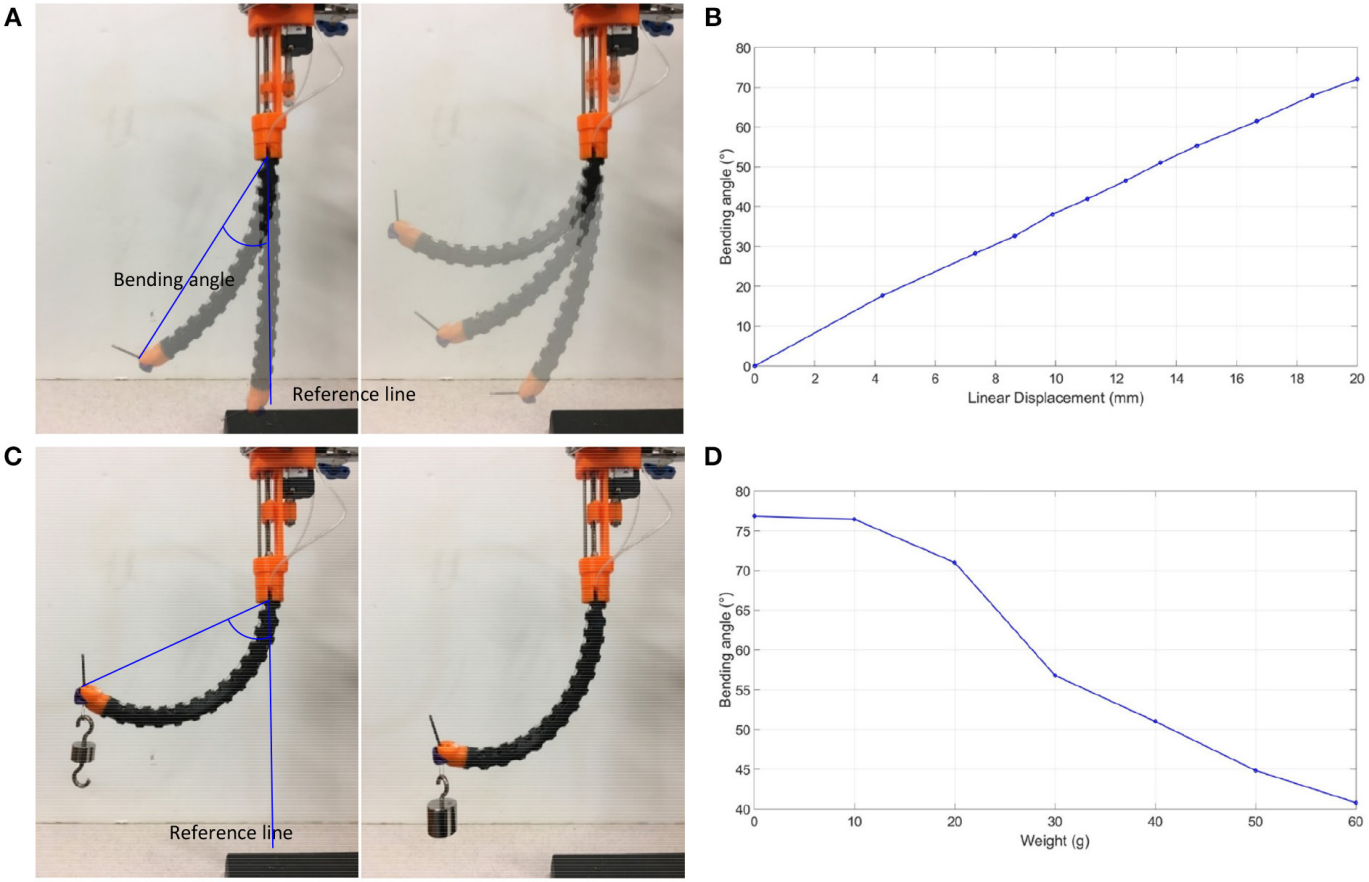
Bending tests. **(A)** Bending angle measurement without loading, **(B)** Bending angle graph without loading, **(C)** Bending angle measurement under loading, **(D)** Bending angle graph under loading.

The prototype was subject to bending without load, starting from a fully extended bending angle/reference line, as shown in Figure 7A. The fiber/optic tendon was controlled randomly in an increasing fashion to see how it corresponded to the bending angle. The slider is managed to pull the tendon to bend the flexible tube. The linear displacement transducer records the displacement of the slider. The displacement of the slide starts from 0 to 20 mm with a 2 mm interval. A camera is positioned perpendicularly to the experimental setup to capture the status of the flexible tube. The images were then uploaded into ImageJ, an open-source image processing program, and the bending angles were measured. The displacements and bending angles were plotted by MATLAB (MATLAB, The Mathworks Inc., Natick, MA, USA). The prototype exhibited an approximately linear relationship between the distance the tendon moved and the bending angle, as shown in Figure 7B. According to this result, we can obtain a certain bending angle by setting the tendon's movement. Next, the prototype was subjected to bending under loads, from 0 to 60 g, starting from a fully extended bending angle/reference line, as shown in Figure 7C. The loads were loaded at the tip and added in intervals of 10 g. Like the previous experiments, images were captured, analyzed by the image processor, and plotted on a graph. As shown in Figure 7D, it was observed that as the weights increase, the bending angle decreases. The relationship between the bending angle and the number of weights loaded at the tip highlights the importance of having the balloon stabilization method incorporated in our prototype. The drilling will bring counterforce on the tip.

### 3.3. Balloon Stability Test

**Figure 8:**
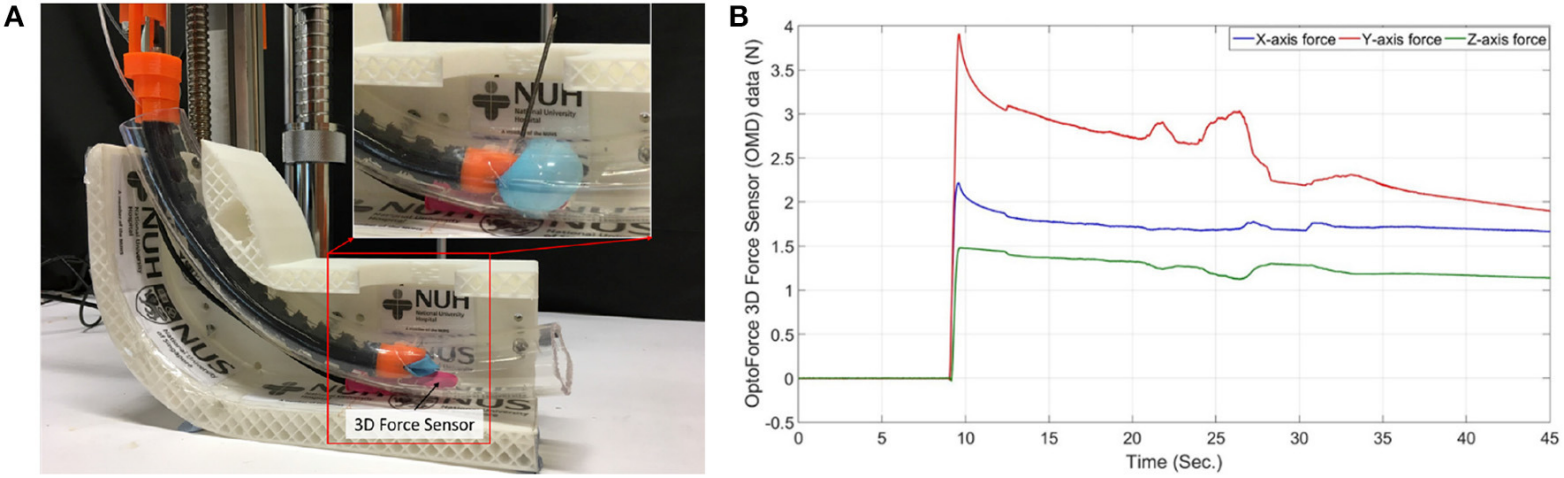
Balloon stability test. **(A)** Balloon expansion force with drilling measurement method, **(B)** Balloon expansion force graph.

The experiment in Figure 8 involves inserting the prototype into a 2.2 cm diameter 20 cm long PVC tube and measuring the 3-dimensional forces produced by the balloon's expansion without drilling and during drilling. A tiny slot was made at the bottom of the transparent PVC tube to fit a 3D force sensor (Resolution: 2.5 mN, OMD-10-SE-10N, Optoforce Ltd.) and to capture the maximum forces exerted by the balloon during inflation. The maximum forces captured in the X-axis, Y-axis, and Z-axis direction during drilling, shown in Figure 8B, were 2.189, 3.916, and 1.496 N, respectively. There was a gradual decline in the balloon's forces along time in the graphs, which is due to loose sealing. However, the fluctuations of the measured expansion forces during the drilling indicate the counterforce's existence. Therefore, a balloon to stabilize the drilling is necessary since it can produce a more accurate drilling process.

### 3.4. Phantom Tests

**Figure 9:**
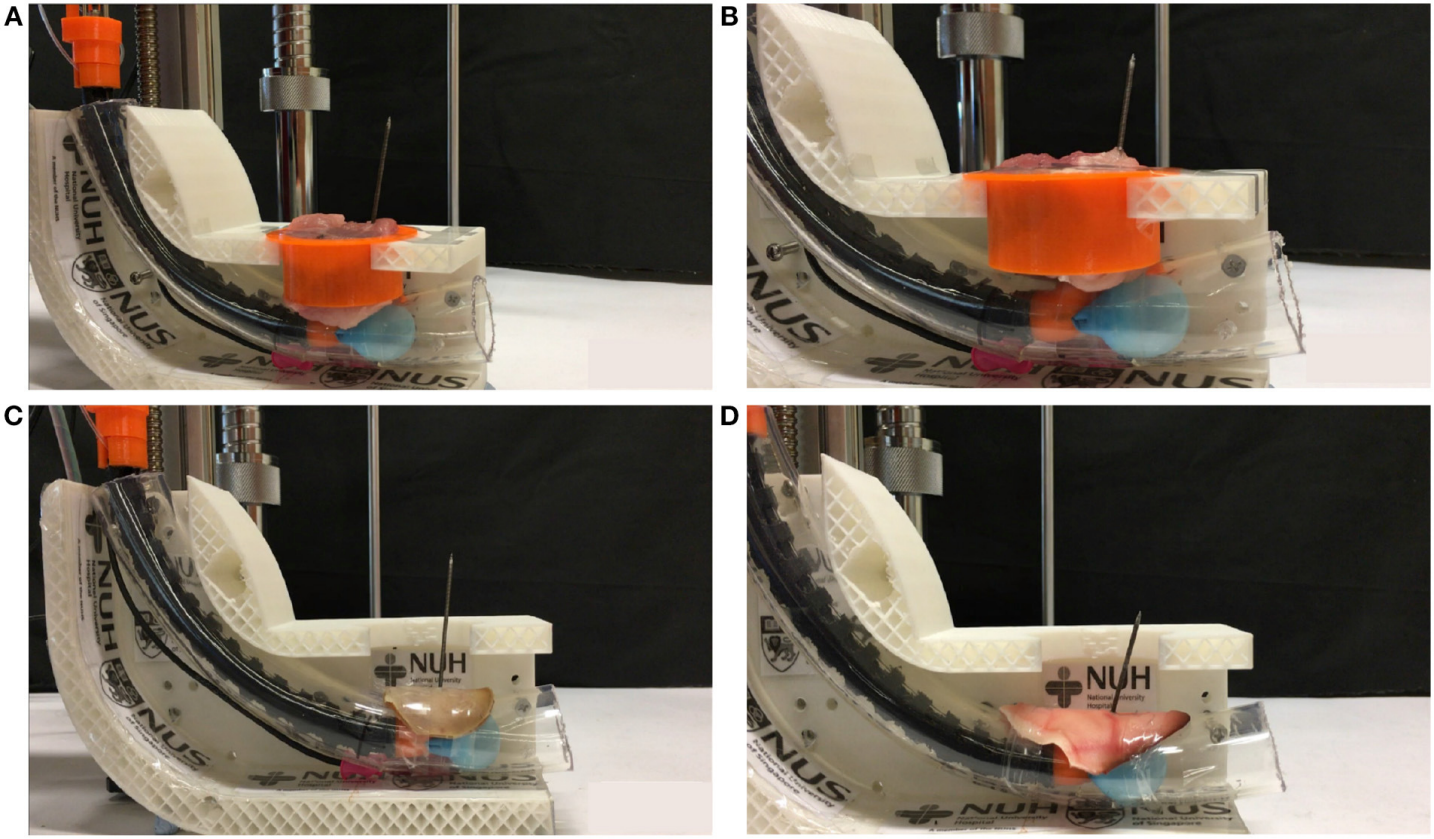
Phantom tests. **(A)** Drilling through porcine lean meat, **(B)** Drilling through porcine lion, **(C)** Drilling through porcine smoked ear cartilage, **(D)** Drilling through porcine raw ear cartilage.

The prototype can drill through various porcine parts to simulate drilling through the human trachea using the same setup as the previous experiment. The parts were porcine loin and lean meat, shown in Figures 9A,B, respectively. Further validation experiments were on the porcine ear cartilage. The human ear's cartilage shares similar cartilage to the pig ear as three parts of the middle ear cavity in humans and pigs are broadly similar. Our prototype can drill through porcine ear cartilage that was smoked and raw, shown in Figures 9C,D, respectively. The prototype was able to pierce through the porcine loin, lean meat, and ear cartilage successfully. The porcine loin & lean meat was the least sturdy, followed by the smoked ear cartilage and raw ear cartilage. Our technology was able to drill through the ear cartilage, which bears a close resemblance to the human trachea.

## 4. Discussion

We had tested our prototype to satisfy the basic requirements and mechanics of performing a trans-oral tracheostomy. The prototype will potentially decrease the time and errors of novice operators in reaching the target anatomical structures. However, as a robotic medical system, some areas could be further optimized and improved. Currently, the control relies on human visual feedback. In the next prototype, closed-loop control of our prototype will be incorporated. Besides, shape and tip-tissue contact force sensing will be introduced, as this will enable us to introduce haptic feedback to the position, and orientate the needle, reducing surgical risks.

The existing air supply tubes could allow the different air entering the balloon, thus allowing our prototype to suit the patients' various applications and anatomical structures. A peristaltic pump could enable the air to inflate and deflate the balloon in a controlled manner and modulate the airflow. Besides, there is no oxygen supply channel in the current prototype, a channel to supply oxygen to the patient during the procedure will be added.

The precision of skin entry from the inside-out approach will be validated in comparative phantom and cadaver experiments. The performance of the prototype performance against conventional PT and OT will be evaluated. We plan to explore imaging techniques, such as ultrasound (US) and computed tomography (CT) imaging in our prototype. Additionally, we will also incorporate needle navigation and trajectory planning techniques into our prototype. These techniques are typically used with image guidance technologies to improve accuracy and efficiency and help preoperative planning and intraoperative planning procedures.

## Data Availability Statement

The raw data supporting the conclusions of this article will be made available by the authors, without undue reservation.

## Author Contributions

HR and CL conceived the project ideas and supervised the project. XX and HP established the experimental hardware setup and recorded the experimental results. MM, HR, CL, and XX were involved in the discussion and manuscript revisions. All authors carried out the experimental validations in the hospital setups and co-wrote the manuscript.

## Conflict of Interest

The authors declare that the research was conducted in the absence of any commercial or financial relationships that could be construed as a potential conflict of interest.
